# Scutellarin Reduces Endothelium Dysfunction through the PKG-I Pathway

**DOI:** 10.1155/2015/430271

**Published:** 2015-10-19

**Authors:** Xiaohua Du, Chen Chen, Min Zhang, Donghua Cai, Jiaqi Sun, Jian Yang, Na Hu, Congji Ma, Liyan Zhang, Jun Zhang, Weimin Yang

**Affiliations:** ^1^School of Pharmaceutical Science and Yunnan Key Laboratory of Pharmacology for Natural Products, Kunming Medical University, Kunming 650500, China; ^2^Department of Respiratory Medicine, First Affiliated Hospital of Kunming Medical University, Kunming 650032, China; ^3^Pharmacy Department, First Affiliated Hospital of Kunming Medical University, Kunming 650032, China; ^4^Department of Clinical Pharmacy, First Affiliated Hospital of Kunming Medical University, Kunming 650032, China

## Abstract

*Purpose*. In this report, we investigated the protective mechanism of scutellarin (SCU) *in vitro* and *in vivo* which could be involved in endothelial cGMP-dependent protein kinase (PKG), vasodilator stimulated phosphoprotein (VASP) pathway, and vascular endothelium dysfunction (EtD). *Method*. Human brain microvascular endothelial cells (HBMECs) with hypoxia reoxygenation (HR) treatment and rats with cerebral ischemia reperfusion (CIR) treatment were applied. Protein and mRNA expression of PKG, VASP, and p-VASP were evaluated by Western blot and RT-PCR methods. Vascular EtD was assessed by using wire myography to determine endothelium-dependent vasorelaxation in isolated rat basilar artery (BA). *Result*. In cultured HBMECs, SCU (0.1, 1, and 10 *μ*M) increased cell viability, mRNA, protein level, and phosphorylative activity of PKG and VASP against HR injury. In HR model of BA, SCU increased protein level of P-VASP. In rat CIR model, wire myography demonstrated that SCU (45 and 90 mg/kg, i.v.) significantly reduced ischemic size by partially restoring the endothelium dependent vasodilation of BA; PKG inhibitor Rp-8-Br-cGMPS (50 *μ*g/kg, i.v.) reversed this protection of SCU in CIR rats. *Conclusion*. SCU protects against cerebral vascular EtD through endothelial PKG pathway activation.

## 1. Introduction

Vascular endothelium has complex physiological functions such as maintaining vascular tone, inhibiting platelet aggregation, reducing endothelial permeability, reducing adhesion molecules expression, and inhibiting vascular smooth muscle cell (VSMC) proliferation [1]. Endothelial cells, in easily damaged anatomical functional interface, are firstly affected by a variety of injuries including ischemia reperfusion (IR), that stimulating effect leads to the damage of structural integrity and reduce of function and then endothelial dysfunction (EtD) occurs. EtD may be an important basis for a number of diseases [1, 2]. Vascular tension adjustment disorders and abnormal expression of adhesion molecules are the main manifests of EtD. Study has shown that IR produces vascular EtD as defined by abrogated endothelium-dependent dilation [2]. In addition, previous study indicated that hypoxia reoxygenation (HR) caused selective inhibition of response to acetylcholine (ACh) in the cerebral arteries [3].

Ischemia reperfusion (IR) leads to tissue injury in various pathophysiological conditions. IR directly affects the vascular wall and luminal surface of blood vessel, causing damages including hemorrhage, capillary plugging, adhesion and infiltration of granulocytes, impaired vascular permeability, and endothelial dysfunction (EtD) [4]. Endothelial cells, while being particularly susceptible to IR injury, play an active role in IR-induced organ damage [5]. EtD reduces perfusion to ischemia areas and thereby exacerbates tissue injury and subsequent damage [6].

cGMP-dependent protein kinase (PKG) is a serine/threonine protein kinase that is activated by cGMP. Accumulating evidences are demonstrating that PKG phosphorylates a number of biologically important targets which are needed to accomplish multiple cellular functions and its dysregulation has been incriminated in many diseases, such as hypertension, atherosclerosis, chronic heart failure, left ventricular hypertrophy, ventricular remodelling, IR injury, diabetes, and cancer [7]. Among the three isoforms of PKG-I*α*, PKG-I*β*, and PKG-II, PKG-I*α* is the strongest vascular tone modulator regulating cell motility, migration, proliferation, and vascular tone [8].

Moreover, there was report that PKG is involved in testosterone-induced vasodilation of human umbilical artery [9]. Upon activation, PKG phosphorylates VASP, which in turn activates downstream ion channels, leading to vascular smooth muscle relaxation and vasodilation. Previous reports have suggested that vasorelaxant effects of baicalin are mainly attributed to voltage-dependent Ca^2+^ channel (VDCC) inhibition and BKCa channel activation through PKA and PKG pathways [10].

Vasodilator stimulated phosphoprotein (VASP) belongs to the Ena/VASP protein family and is an important PKG-I substrate and actin regulatory protein. Studies [11] suggested that phosphorylated VASP at serine 239 (p-VASP) has been shown to be a useful monitor for PKG-I activity in intact cells.

Scutellarin (SCU), 4′,5,6-trihydroxy flavonoid-7-glucuronide, was reported to be the primary active ingredient of breviscapine, which is a mixture of flavonoid glycosides isolated from a Chinese traditional medicine plant* Erigeron breviscapus* (Vant.) Hand. Mazz. [12]. The plant extracts and SCU have been used in China to treat a variety of disorders including cardiovascular, cerebrovascular, and inflammatory diseases for many years [13]. In animal studies, SCU has been reported to be neuroprotective in rat cerebral ischemia reperfusion (CIR) models [14] via augmentation of antioxidant defense capacity [13]. In addition, SCU prevented EtD in diabetic rats and inhibited translocation of protein kinase C in diabetic thoracic aorta of the rat [15]. Our earlier study [16, 17] showed that relaxation effect of SCU on artery was predominantly endothelium dependent and partially involved the catalase-sensitive nitric oxide synthase signaling pathway.

Based on these observations, we hypothesize that SCU reduces EtD through the PKG-I pathway. To verify this hypothesis, we test the protein level and mRNA expression of PKG-I, VASP, and p-VASP in human brain microvascular endothelial cells (HBMECs). The effects of SCU on EtD of brain basilar artery (BA) and infarct size were checked in rats with CIR injury.

## 2. Materials and Methods

### 2.1. Chemicals and Drugs

SCU was obtained from Kunming Longjin Pharmaceutical Co. (Kunming, China). Cell culture reagents DMEM, modified RPMI-1640 medium, and fetal bovine serum were obtained from the Hyclone (Thermo Scientific, USA). Other items include Wire Myograph System DMT (Danish Myo Technology Company, Denmark) and Power Lab data recording and analytical system (ADInstruments Ltd., Australia). HBMECs were purchased from Yangsen Biology Limited Company (Shanghai, China). U46619 was purchased from Cayman Chemical Company. PKG inhibitor Rp-8-Br-cGMPS was purchased from Santa Cruz Biotechnology (Dallas, TX, USA). 3-(4,5-Dimethylthiazol-2-yl)-2,5-diphenyltetrazolium bromide (MTT), triphenyl tetrazolium chloride (TTC), and ACh were purchased from Sigma-Aldrich (St. Louis, MO, USA).

### 2.2. Animals

Sprague-Dawley rats (180–220 g, male and female in each half) were provided by the animal center of Kunming Medical University. All animals were housed in microisolation under conditions of constant temperature and controlled illumination (light on 12-hour light/dark cycle). Food and water were available ad libitum. All the animals used in the experiment received humane care. All surgical and experimental procedures were in accordance with the institutional animal care guidelines. The animal study was approved by the Animal Care and Use Committee of Kunming Medical University and conformed to the standards set by the Yunnan Experimental Animal Management Board.

### 2.3. Methods

#### 2.3.1. Endothelial Cell Culture and HR Treatment

HBMECs were obtained from the Shanghai Yangsen Biochemical Technology Company (Shanghai, China) and grown in 1640 medium supplemented with 10% fetal bovine serum and 1% penicillin/streptomycin antibiotics. Tightly confluent monolayers of HBMECs from 4th−15th passage were used in all experiments. In experiments checking the effects of SCU under normal condition, cells were treated with vehicle control (NS) or SCU (0.1, 1.0, and 10.0 *μ*M) at different concentrations for 26 hours. In experiments with HR treatments, cells were divided into 5 groups including control, HR model, HR + SCU 0.1 *μ*M, HR + SCU 1 *μ*M, and HR + SCU 10 *μ*M group. Control cells were cultured in parallel and kept in normal culture condition for the entire time period (26 h). Simulated HR injury was induced according to previously described procedures [18] with minor modifications. Briefly, HBMECs were placed in a humidified hypoxic chamber (HF100, Heal Force Biotech Co., Shanghai, China) for 12 h of hypoxia (5% CO_2_ + 2% O_2_ + 93% N_2_) with medium free of glucose and serum at 37°C, followed by 12 h of reoxygenation (5% CO_2_ + 95% air) in complete medium containing glucose and serum. For HR + SCU groups, cells were incubated with SCU at different concentrations for 2 h prior to hypoxia treatment and during hypoxia (12 h) and reoxygenation (12 h) injury. For control group, cells were treated with vehicle control (NS) for 26 h, and for SCU groups, cells were given different concentrations of SCU for 26 h separately. At the end of the experiment, cell viability was examined using MTT assay as described below. Furthermore, cells of each group were also collected for RT-PCR assay and Western blotting assay as described in the following sections. All experiments were performed in triplicate.

#### 2.3.2. MTT Assay of Cell Viability

For MTT assay, cells were plated in 96-well flat-bottomed plates at a density of 3 × 10^4^ cells/mL and 90 *μ*L/well. Cells were cultured in normal culture condition or treated with simulated HR injury as described above. After the treatments, 20 *μ*L of MTT (5 mg/mL) was added to each well and the plates were incubated for 4 h at 37°C. Then, 100 *μ*L of lysis buffer (20% sodium dodecyl sulfate [SDS] in 50% N,N-dimethylformamide, containing 0.4% [v : v] 1 N HCL and 0.5% [v : v] 80% acetic acid) was added to each well and incubated overnight. Cell viability was determined by measuring the ability of metabolically active cells to convert the yellow tetrazolium salt MTT (5 mg/mL, PH = 7.4) into purple formazan crystals with a microplate reader at 570 nm. Results of three independent experiments (each conducted in triplicate) were used for statistical analysis.

#### 2.3.3. Western Blot Analysis

The PKG-I, VASP, and p-VASP protein levels in lyzed cell were examined by Western analysis. Protein concentrations were determined by using BCA Protein Assay Kit (Beyotime Biotechnology, Haimen, Jiangsu, China). Total protein (20 *μ*g) was fractionated on 10% SDS-PAGE and transferred to nitrocellulose membranes. Membranes were blocked with 10% defatted milk powder solution at room temperature for 2 h and incubated overnight at 4°C with the rabbit antibodies against VASP (concentration 1 : 500, Cell Signaling Technology, Inc., USA), p-VASP (Ser239) (concentration 1 : 2000, Santa Cruz Biotechnology, USA), and PKG-I (concentration 1 : 1000, Santa Cruz Biotechnology, USA). After three washes, membranes were incubated with horseradish peroxidase conjugated goat anti-rabbit IgG antibody (Santa Cruz Biotechnology, USA) for 1.5 h at room temperature. Then four washes were repeated, and the protein was visualized with enhanced chemiluminescence kit (Sigma, USA). The density values of bands were quantified by densitometric analysis of scanned images (Scion Image 4.03). The relative protein ratio was calculated by determining the integrated intensity of the bands of each treated group as a ratio of the control condition.

#### 2.3.4. RT-PCR

Total RNAs were isolated from the cells by using Trizol reagent (TaKaRa, Japan). The sequences of primers (human PKG-I*α* and GAPDH) used in this study were PKG-I*α* (forward, AGCGGATCGAAGCAGGAGGC and reverse, TGACGGTCGCTGTCC GGGTA, 728 bp) and GAPDH (forward, AATCCC ATCACCATCTTCC and reverse, GAGTCCT TCCACGATACCAA, 309 bp), respectively.

Total RNA (1 *μ*g) was reverse-transcribed into cDNA using a Quantscript RT Kit (Tiangen, China). PCR was performed using a PCR MasterMix Kit (BioTeke, China) in a GeneAmp PCR system 9600 (ABI Int.). cDNA was amplified under the thermocycling conditions as follows: 3 min initial denaturation at 94°C (1 cycle), 30 s denaturation at 94°C (35 cycles), 30 s annealing at 57°C for human PKG and VASP, and 45 s extension at 72°C. The last amplification was followed by a final 7 min incubation at 72°C. PCR products were separated by electrophoresis through 1% agarose gel, stained with ethidium bromide, and visualized by UV transillumination in a Tocan Gel Imaging System (Tocan Co., Shanghai, China). GAPDH was used as an internal control. The mRNA level was calculated by determining the integrated intensity of the bands of each treated group as a ratio of the control. Each sample was measured in triplicate and the mean threshold cycle (Ct) value was calculated.

#### 2.3.5. Rat CIR Model and Evaluation of Cerebral Infarct Volume

At room temperature (22 ± 2°C) conditions, the rats with 10% chloral hydrate (0.035 mL/kg) intraperitoneal injection of anesthesia were supinely fixed on the operating table, giving the tail vein injection of drugs while starting surgery. The right external carotid artery was ligated, and then right middle cerebral artery was given reperfusion 24 h after occlusion for 1 h [19]. At the end of the reperfusion, rats were decapitated. The brains were rapidly removed and frozen immediately at −20°C for 15 min, and then the brains were cut into 2 mm thick coronal sections which were stained with 1% TTC at 37°C for 10 min followed by fixation with 4% paraformaldehyde for 1 hour. Unstained areas were defined as ischemic lesions, whereas normal tissue was stained red. The infarct areas were traced and quantified with IPP software. Infarct areas of all sections were added to derive the total infarct area, which was multiplied by the thickness of the brain sections to obtain the infarct volume. To compensate for the effect of brain edema, the corrected infarct volume was calculated as previously described [20]. Corrected infarct volume equals total infarct volume multiply contralateral hemisphere volume/ipsilateral hemisphere volume.

To evaluate the effects of SCU on CIR injury, SD rats were divided into four groups (*n* = 10–12 in each group): sham, CIR model, and two SCU groups (45 or 90 mg/kg, i.v.). In another experiment, the influence of PKG inhibitor on the effects of SCU was assessed. The rats were divided into four groups (*n* = 10–15 each): CIR model, SCU (90 mg/kg, i.v.), PKG inhibitor (50 *μ*g/kg, i.v.), and SCU (90 mg/kg, i.v.) + PKG inhibitor (50 *μ*g/kg, i.v.) treated group. Drug infusion was initiated intravenously during surgery via the tail vein (2 mL/h). The sham and CIR model groups were given normal saline (NS).

#### 2.3.6. Evaluation of EtD in Isolated BA from CIR Rats

The above treated rats were sacrificed and the BA was gently excised and rinsed off blood in 4°C PSS buffer solution (140 mM NaCl, 4.7 mM KCl, 1.6 mM CaCl_2_, 1.2 mM MgSO_4_, 1.2 mM MOPS, 1.4 mM Na_2_HPO_4_, 0.02 mM EDTA, and 5.6 mM D-glucose. The PH of PSS was preadjusted to 7.4 at 37°C). The BA was cleaned of fat and surrounding tissue, and then was cut into approximately 1-2 mm rings.

An integrated wire myograph system (Model 620 M, DMT Asia Ltd., Shanghai, China) was applied with a Motic SMZ168-TL stereomicroscope to mount BA rings on 60 *μ*m steel wires in separated tissue baths of the wire myograph system for tension recording. The tissue baths were filled with PSS solution (PH 7.4) at 37 ± 1°C and aerated with O_2_. Washout was performed by draining and replacing the bathing solution using a syringe. Isometric tension signals were recorded and data were collected by a PowerLab data acquisition system (ADInstruments Asia, Shanghai, China). Each ring was stretched to an optimal tension of 1 mN and permitted to equilibrate for 90 minutes before the experiment started. The rings were contracted by U46619 (1 *μ*mol/L) and relaxed using cumulative addition of ACh (0.001–1000 *μ*mol/L) to test the endothelial-dependent vasodilation by calculating EC_50_ and *E*_max_ values.

#### 2.3.7. Preparation of HR Model of Isolated BA and Treatment with SCU

Rats were sacrificed by intraperitoneal injection of 10% (0.1 mL/100 g) urethane. BA was removed and dissected free from brain tissues. Isolated BA segments were placed in anaerobic sugar-free physical salt solution (PSS) with 3-(N-morpholino)propanesulfonic acid sodium polystyrene sulfonate (MOPS), which leads to nitrogen hypoxia for 2 hours; then the BA segments were changed into normal MOPS-PSS solution with restoring oxygen and glucose for 2 hours. During HR damage, SCU (50 *μ*M, 100 *μ*M) was incubated along in SCU groups, while HR model group was given vehicle (NS). Control group was the normal BA. At the end of HR injury, BA transferred into a precooled glass homogenizer containing 200 *μ*L precooled RIPA buffer (50 mM Tris-HCl, 150 mM NaCl, 1 mM EDTA, 1 mM PMSF, 1 mM Na_3_VO_4_, 1 mM NaF, 1% NP-40, 0.25% Na-deoxycholate, 10% glycerol, and 1 mg/mL of each of the phosphatase inhibitors aprotinin, leupeptin, and pepstatin, PH 7.4). Vessel tissues were then homogenized for 10 min, transferred to a 1.5 mL Eppendorf tube, and centrifuged at 18,000 rpm for 10 min at 4°C. The supernatant was collected by decantation and stored at −80°C until Western blot analysis.

#### 2.3.8. Calculations and Statistical Analysis

Data were expressed as means ± SEM. Statistical analysis was performed using statistical software Sigma Stat 3.5. The *E*_max_ value represents the maximal vasodilative response that drug caused; EC_50_ is the concentration in which drug makes 50% of the maximum vasodilative effect. Nonlinear regression analysis for individual concentration-response curves was performed using a Hill algorithm in Sigma Plot 10.0, allowing for an individual geometric “EC_50_” value to be calculated. *E*_max_ = [(the maximal stress of precontraction − the minimal stress)/the maximal stress of precontraction] *∗* 100%. Comparisons were made using one-way ANOVA analysis. *P* < 0.05 was considered statistically significant.

## 3. Results

### 3.1. Effect of SCU on PKG-I and Cell Viability in Normal Cultured HBMECs

**Figure 1:**
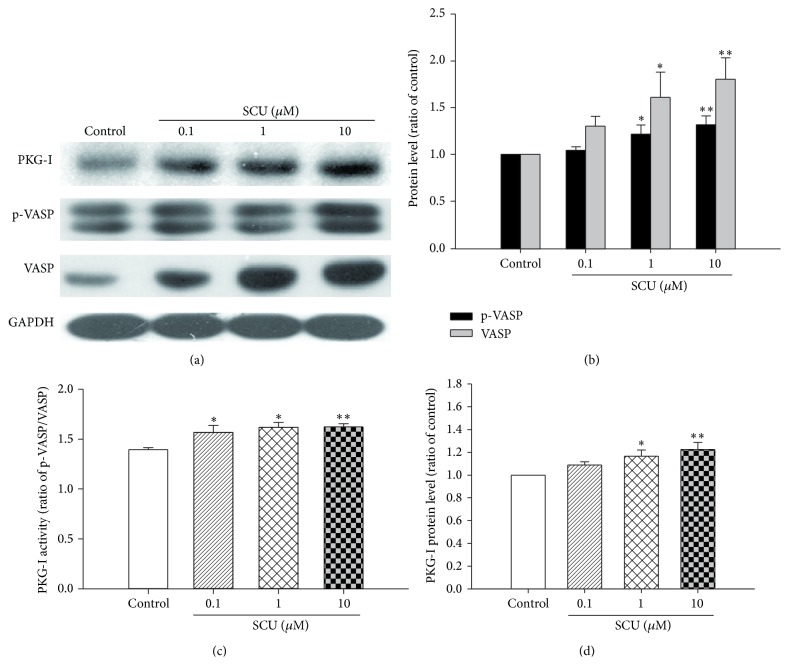
The effect of SCU on the protein level of PKG-I, VASP, and p-VASP protein in normal HBMECs. (a) Representative immunoblot and quantification of PKG-I, p-VASP, and VASP protein expression in normal HBMECs with SCU treatment. (b) The ratio of p-VASP and VASP compared with control in normal HBMECs with SCU treatment. Protein ratio of control was calculated by determining band integrated intensity as ratio of control. (c) The ratio of p-VASP compared with VASP in normal HBMECs with SCU treatment. (d) The ratio of PKG-I compared with control in normal HBMECs with SCU treatment. Protein ratio of control was calculated by determining band integrated intensity as ratio of control. Normal HBMECs, under normal culture condition: cells were incubated with SCU (0.1, 1, and 10 *μ*M) for 24 h except control group. One-way ANOVA on Rank followed by SNK test, ^*∗*^ *P* < 0.05, ^*∗∗*^ *P* < 0.01, compared to control. Data are means ± SEM; *n* = 3 independent experiments with independent culture.

As seen from Figures 1(a), 1(b), and 1(d), SCU (0.1, 1 and 10 *μ*M) treatment increased the protein levels PKG-I, p-VASP, and VASP in normal HBMECs compared with control group. In addition, the ratio of p-VASP to VASP that indicated the activity of PKG was also increased under SCU treatment (Figure 1(c)). Particularly, there was apparent increase in protein levels of PKG-I, P-VASP, VASP, and PKG activity in normal HBMECs with SCU (1 and 10 *μ*M) treatment.

**Figure 2:**
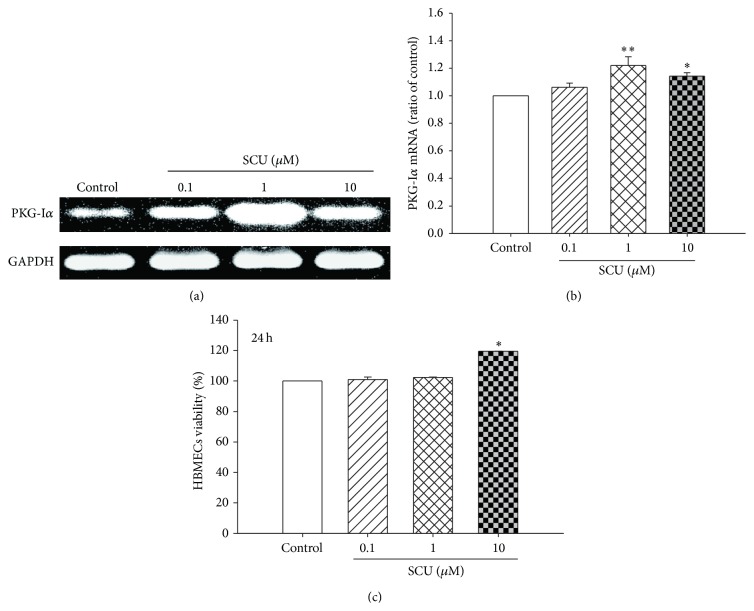
The effect of SCU on mRNA expression of PKG-I and cell viability in normal HBMECs. (a) Representative electrophotogram and quantification of PKG-I*α* mRNA expression in normal HBMECs. (b) The mRNA ratio of PKG-I*α* in normal HBMECs. Ratio of control was calculated by determining band integrated intensity as ratio of control. (c) The ratio of cell viability compared with control in normal HBMECs with SCU (0.1, 1, and 10 *μ*M) treatment. Cell viability was examined using MTT assay. Normal HBMECs, under normal culture condition: cells were incubated with SCU (0.1, 1, and 10 *μ*M) for 24 h except control group. One-way ANOVA on Rank followed by SNK test, ^*∗*^ *P* < 0.05, ^*∗∗*^ *P* < 0.01, compared to control. Data are means ± SEM; *n* = 3 independent experiments with independent culture.

As shown in Figures 2(a) and 2(b), under normal condition, SCU (0.1, 1, and 10 *μ*M) incubation increased mRNA level of PKG-I*α*, especially in SCU 1 *μ*M group.

In our preliminary experiments checking the influence of SCU on cell viability of normal HBMECs, SCU with dose < 0.1 *μ*M did not cause significant changes in cell viability while SCU with dose >100 *μ*M exhibited cytotoxicity (data not shown). Therefore, SCU at doses of 0.1, 1, and 10 *μ*M was used in the present study. As shown in Figure 2(c), results of MTT assay indicated that SCU 10 *μ*M incubation raised apparently cell viability under normal culture condition, while there are not significant changes in SCU 0.1 and 1 *μ*M group.

### 3.2. Effect of SCU on PKG-I and Cell Viability in HBMECs with HR Treatment

**Figure 3:**
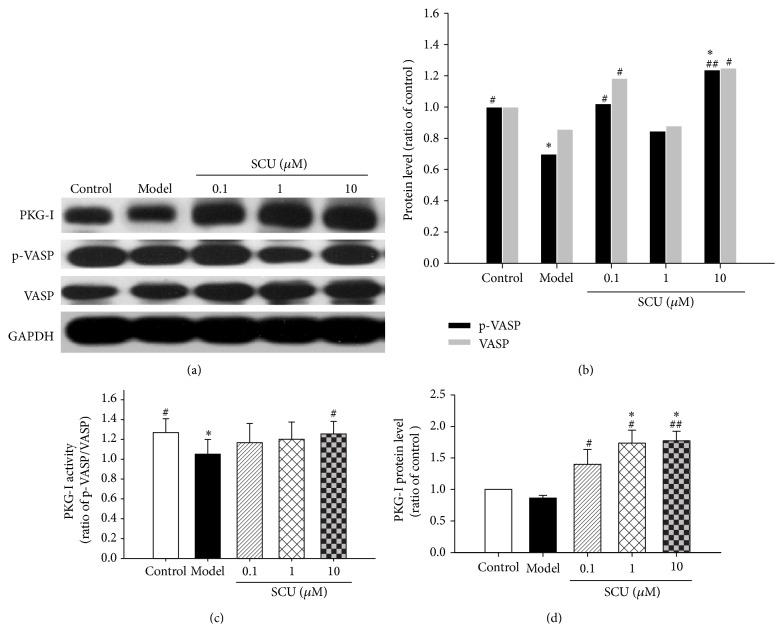
The effect of SCU on the protein level of PKG-I, VASP, and p-VASP in HR HBMECs. (a) Representative immunoblot and quantification of PKG-I, p-VASP, and VASP protein expression in HR HBMECs with SCU treatment. (b) The ratio of p-VASP and VASP compared with control in HR HBMECs with SCU treatment. Protein ratio of control was calculated by determining band integrated intensity as ratio of control. (c) The ratio of p-VASP compared with VASP in HR HBMECs with SCU treatment. (d) The ratio of PKG-I compared with control in HR HBMECs with SCU treatment. Protein ratio of control was calculated by determining band integrated intensity as ratio of control. Control group: cells were treated with vehicle control (NS) under normal culture condition for 26 h. Model group: cells were incubated under normal culture condition for 2 h and then given HR treatment (hypoxia 12/reoxygenation 12 hours). SCU groups: cells were incubated with SCU for 2 h prior to HR injury. One-way ANOVA followed by SNK test, ^#^ *P* < 0.05, ^##^ *P* < 0.01, compared to model of HR group, ^*∗*^ *P* < 0.05, ^*∗∗*^ *P* < 0.01, compared to control group. Data are means ± SEM; *n* = 3 independent experiments with independent culture.

The effects of SCU on protein expression of PKG-I, VASP, and phosphorylation of VASP (p-VASP) under HR injury were shown in Figure 3. Compared with control, HR injury decreased p-VASP, VASP, and PKG, especially p-VASP; compared with model, SCU preincubations could significantly raise the protein expression of p-VASP, VASP, and PKG-I (Figures 3(a), 3(b), and 3(d)). HR injury also reduced PKG activity in model group (ratio of p-VASP and VASP) while SCU pretreatment could significantly augment it, especially in SCU 10 *μ*M (Figure 3(c)).

**Figure 4:**
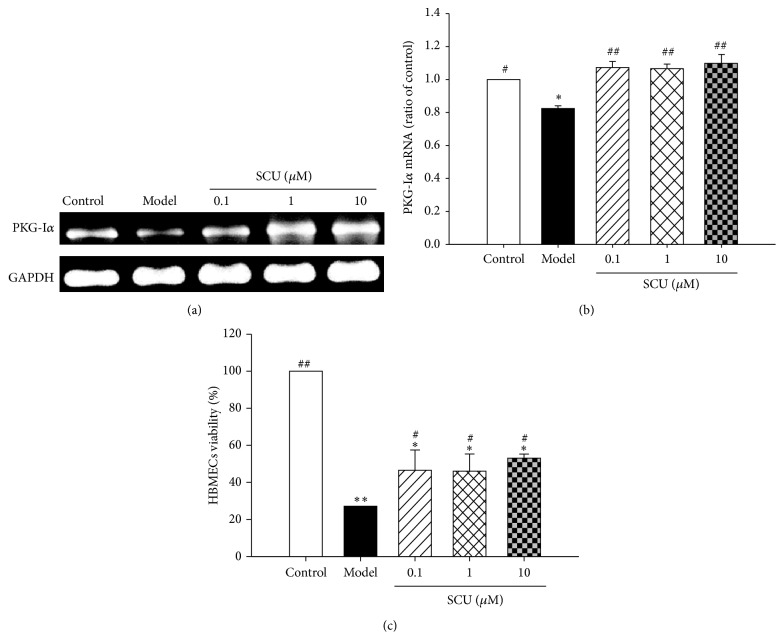
The effect of scutellarin (SCU) on mRNA expression of PKG and cell viability in HR HBMECs. (a) Representative electrophotogram and quantification of PKG-I*α* mRNA expression in HR HBMECs. (b) The mRNA ratio of PKG-I*α* in HR HBMECs. Ratio of control was calculated by determining band integrated intensity as ratio of control. (c) SCU increases cell viability in hypoxia reoxygenation- (HR-) treated human brain microvascular endothelial cells (HBMECs). Cell viability was examined using MTT assay and expressed as ratio of control. Control group: cells were treated with vehicle control (NS) under normal culture condition for 26 h. Model group: cells were incubated under normal culture condition for 2 h and then given HR treatment (hypoxia 12/reoxygenation 12 hours). One-way ANOVA followed by SNK test, ^#^ *P* < 0.05, ^##^ *P* < 0.01, compared to model of HR group, ^*∗*^ *P* < 0.05, ^*∗∗*^ *P* < 0.01, compared to control group. Data are means ± SEM; *n* = 3 independent experiments with independent culture.

As seen from Figures 4(a) and 4(b), the expression of PKG-Ι*α* mRNA was significantly reduced in HR model group, while SCU (0.1, 1, and 10 *μ*M) pretreatment increased it sharply (Figure 4(b)). The results indicated SCU could upregulate mRNA expression of PKG-I*α* and antagonized the HR-induced injury.

As shown in Figure 4(c), HR injury greatly reduced cell viability of HBMECs, and pretreatment of SCU (0.1, 1, and 10 *μ*M) could protect cells from HR-induced injury, especially the obvious effect of SCU (10 *μ*M) on viability (Figure 4(c)). The data indicated that SCU has the protective effects on HBMECs against HR injury.

### 3.3. The Effect of SCU and PKG Inhibitor in Rats with CIR Treatment

**Figure 5:**
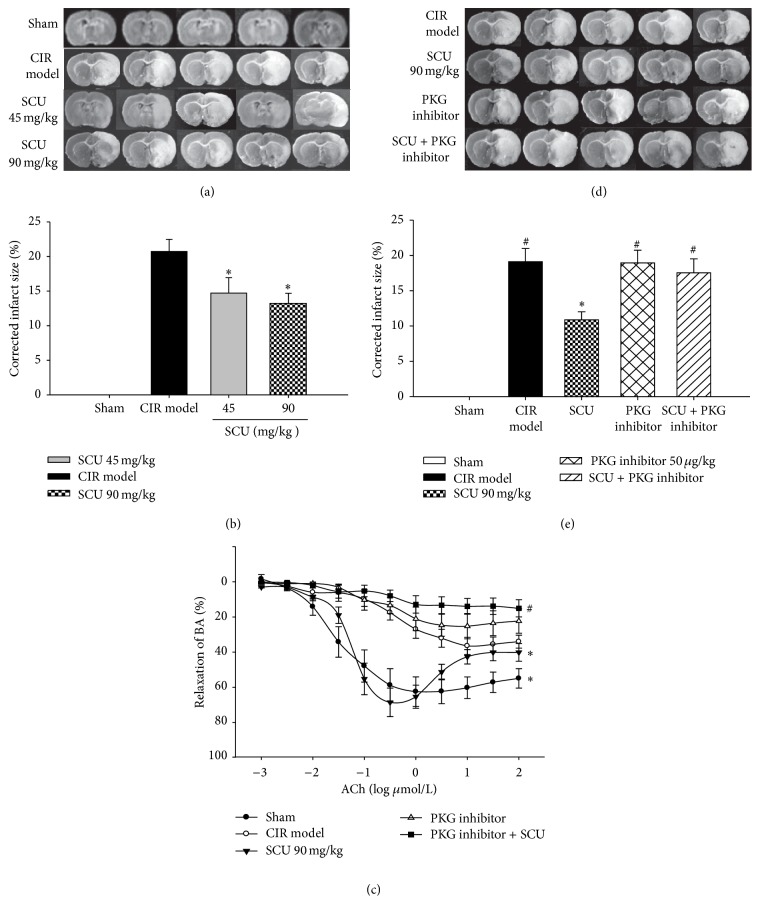
The effect of SCU and PKG inhibitor in rats with CIR treatment. (a) Representative images of triphenyl tetrazolium chloride (TTC) staining of ischemic brain slices from CIR (ischemia 60 minutes/reperfusion 24 hours) rats with SCU treatment. (b) Quantification of the corrected cerebral infarct volume in CIR rats with SCU treatment. *n* = 10 rats in each group. One-way ANOVA followed by SNK test versus model, ^*∗∗*^ *P* < 0.01, ^*∗∗∗*^ *P* < 0.001. (c) Cumulative concentration response curves of acetylcholine chloride (ACh) in isolated basilar artery (BA) segments from normal or CIR rats which were given SCU (90 mg/kg, i.v.) or PKG inhibitor treatment. Relaxation in response to ACh is expressed as a percent of precontraction with U46199 (1 *μ*mol/L). *n* = 8 segments obtained from 3 rats with different treatments. Two-way ANOVA test versus model, ^*∗*^ *P* < 0.05, versus SCU, ^#^ *P* < 0.05. (d) Representative images of TTC staining of ischemic brain slices from CIR (ischemia 60 minutes/reperfusion 24 hours) rats, with SCU (90 mg/kg i.v.) and PKG inhibitor (Rp-8-Br-cGMPS, 50 *μ*g/kg i.v.) alone and combined (SCU 90 mg/kg + Rp-8-Br-cGMPS 50 *μ*g/kg) treatments. (e) Quantification of the corrected cerebral infarct size in CIR rats administered with SCU and PKG inhibitor. *n* = 10 rats in each group. One-way ANOVA followed by SNK test versus model, ^*∗*^ *P* < 0.05, versus SCU, ^#^ *P* < 0.05. Data are means ± SEM.

As seen from Figures 5(a) and 5(b), compared with model group, SCU (45, 90 mg/kg) attenuated infarct size in rats with CIR treatment, especially in SCU 45 mg/kg group. Moreover, the values of vascular tension were reduced in SCU (90 mg/kg) group (Figure 5(c)), while the values of vascular tension in SCU (45 mg/kg) have no significant change.

There was similar change in the values of BA cumulative-response curves of ACh, EC_50_, and *E*_max_ in CIR rats. CIR injury caused BA EC_50_ and *E*_max_ to be higher, while in CIR rats handled with SCU (90 mg/kg) pretreatment, the values of BA cumulative-response curves of ACh, EC_50_, and *E*_max_ were low apparently. These above results showed that SCU (90 mg/kg) pretreatment has a protective effect on vascular EtD induced by CIR, and endothelium-dependent vasodilation in response to ACh was significantly impaired in BA exposed to CIR treatment, which showed that CIR model was successfully made.

In order to check the mechanism of SCU, the PKG inhibitor was used before the CIR rats were given SCU treatment. The results showed that PKG inhibitor reversed the effects of SCU on improving infarct size and BA values of EC_50_ and *E*_max_ in CIR rats dealt with SCU. As presented in Figures 5(d) and 5(e), infarct sizes in rats treated with SCU and PKG inhibitor were bigger than those in rats handled with SCU alone. Meanwhile, similar changes occurred in the EC_50_, *E*_max_, and the values of BA cumulative-response curves of ACh in CIR rats handled with SCU and PKG inhibitor (Figure 5(c) and Table 1). The results indicated that PKG inhibitor reversed the effect of SCU on BA of rats with CIR treatment through inhibiting improvement of endothelium vasodilation and suggested that SCU might protect against CIR via PKG signal pathway.

### 3.4. The Effect of SCU on p-VASP in Isolated BA of Rats with HR Treatment

**Figure 6:**
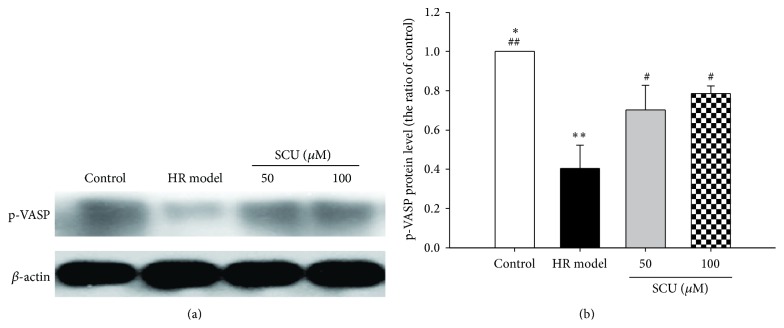
The effect of SCU on p-VASP in isolated BA of rats with HR treatment. (a) Representative immunoblot and quantification of p-VASP protein expression in rat BA with CIR and SCU treatment. (b) The ratio of p-VASP compared with control and CIR model in BA of rats with treatment of SCU and CIR injury. Protein ratios of control and model were calculated by the ratio of each bar compared to the control ratio and to the model ratio separately after the band integrated intensity as ratio of internal control (*β*-actin). *n* = 8 rats in each group. One-way ANOVA followed by SNK test versus control, ^*∗*^ *P* < 0.05, ^*∗∗*^ *P* < 0.01, versus model, ^#^ *P* < 0.05, ^##^ *P* < 0.01. Data are means ± SEM.

The effects of SCU on protein expression of phosphorylation of VASP (p-VASP) under HR injury were shown in Figures 6(a) and 6(b). HR injury significantly decreased p-VASP while SCU treatment could significantly increase the protein level of p-VASP. This shows that SCU can fight against HR injury in isolated BA blood vessel involved in PKG pathway.

## 4. Discussion

This study advances not only our understanding about the regulation of VASP and PKG-I, but also a mechanism underlying the beneficial effects of SCU in treating cerebrovascular diseases. In the present study, we found firstly, to our knowledge, that SCU increased the protein and mRNA expression of PKG and VASP in HBMECs with HR treatment. Moreover, compared with model group, SCU increased the cell viability and simultaneously raised the viability of PKG-I in HBMECs with HR treatment. This suggested the protective effects of SCU on the injury of HBMECs with HR involved in PKG-I/VASP signaling pathway. In our cell experiment, we found that the effect of SCU on normal HBMECs is sharply weaker than that of HBMECs with HR treatment; this result showed that SCU could fight against HR injury.

In CIR rats, SCU preincubations could significantly decrease brain infarct size, vascular tension, and the values of BA cumulative-response curves of ACh, EC_50_, and *E*_max_ and increase protein level of p-VASP in HR BA. These above results showed that SCU (90 mg/kg) pretreatment has a protective effect on vascular EtD induced by CIR, and endothelium-dependent vasodilation in response to ACh was significantly impaired in BA exposed to CIR treatment, which showed that CIR model was successfully made. Moreover, our results showed that SCU decreased the cerebral infarct size; this is consistent with the previous studies by Lin et al. [21, 22].

In order to check the mechanism of SCU, the PKG inhibitor was used before the CIR rats were given SCU treatment. The result shows that PKG inhibitor reversed the effects of SCU on improving infarct size, vascular tension, and the BA values of EC_50_ and *E*_max_ in CIR rats dealt with SCU. This result indicated that PKG inhibitor reversed the effect of SCU on BA of rats with CIR treatment through inhibiting improvement of endothelium vasodilation and suggested that SCU protects against CIR partially via PKG signal pathway.

Endothelial cells play an important role in controlling local vascular tension. In this study, we proposed one of the mechanisms of SCU-attenuated EtD induced by IR via PKG-I/VASP signaling pathway. Our results showed SCU attenuated the EC_50_ and *E*_max_ values of ACh in isolated BA induced by CIR, but the PKG inhibitor reversed the effects of SCU on improving EC_50_ and *E*_max_ values of BA in CIR rats. This confirmed our hypothesis that SCU attenuated EtD induced by IR via PKG-I/VASP signaling pathway and supported our group's previous studies that SCU vasorelaxation was predominantly endothelium dependent and involved nitric oxide synthase signaling pathway [17]. Meanwhile, we found that PKG inhibitor blocked the effect of SCU on reducing the cerebral infarction size. This mechanism contributes, at least in part, to elucidating the beneficial effects of SCU in CIR injury via PKG-I signal pathway.

SCU may exert its protection effects in IR by preventing generation of ROS, directly scavenging ROS or indirectly through enhancement of cellular antioxidant enzymes [23, 24]. Moreover, SCU could promote angiogenesis in endothelial cells [25], inhibit the apoptosis and the apoptosis inducing factor pathway [26–28], and attenuate mucus production* in vitro* and* in vivo* involving the inhibition of PKC-ERK signaling pathway [29]. SCU benzyl ester has a remarkable protective effect against myocardial ischemic injury and the protective mechanism may associate with its antiapoptotic effect by inhibiting cytochrome C release and caspase-3 activation and attenuate inflammation [30], antitumor [31, 32], antiviral [33], and neuroprotective effect [24]. Long-term administration of SCU improved the cardiac function of MI rats by inhibiting interstitial fibrosis, and the mechanisms may involve the suppression of profibrotic cytokine TGF*β*1 expression and inhibition of p38 MAPK and ERK1/2 phosphorylation [22]. SCU exerts protective effects against IR injury through inhibiting PKC [15, 34]. However, the current study demonstrates that the PKG/VASP pathway plays an important role in pharmacological studies of SCU* in vivo* combining* in vitro* IR model. This observation may have further pharmacy implications because it may contribute to the clarification of the mechanisms behind the observed decreases in cardiocerebral vascular morbidity and mortality in patients receiving SCU or other flavonoids. Studies in another flavonoid baicalin suggest that vasodilator properties were attributed to endothelium-dependent relaxation through the PKG pathway [10]. There are evidences suggesting that ischemia followed by reperfusion causes local dysfunction including EtD [35]. Hypoxia and reoxygenation are two essential elements of ischemia and reperfusion injury. HR may cause different forms of vascular injury, such as hemorrhage, change in vascular permeability, and EtD, including impaired endothelium-dependent vasodilation. Our study showed that IR caused EtD of BA with CIR, and SCU had protective effect on the EtD of BA* in vivo*. This observation was also in accordance with our previous result that SCU could relax isolated aortic rings of rat [17].

The roles of PKG-I isozymes have been documented in many processes including gastrointestinal motility, blood flow, neuronal plasticity, erectile function, lower urinary tract functions, endothelial permeability, and cardiac protection [7, 36–38]. Moreover, there was report that PKG is involved in testosterone-induced vasodilation of human umbilical artery [9]. Upon activation, PKG phosphorylates VASP, which in turn activates downstream ion channels, leading to vascular smooth muscle relaxation and vasodilation. Previous reports have suggested that cGMP/PKG/ROS/calmodulin/CaMKII signaling pathway may regulate cardiomyocyte excitability by opening K_ATP_ channels and contribute to cardiac protection against IR injury [39]. In vascular endothelial cells, PKG-I regulates cell motility, migration, and proliferation. These functions are reported to be essential for vascular permeability and angiogenesis. Experiments with PKG-I deficient vascular model systems have recently established that NO donor-induced VASP phosphorylation is primarily mediated by PKG-I [11]. Our result showed that the actions of PKG-I/VASP signaling may be a novel therapy target for IR injury of BA.

Among the downstream targets of PKG is vasodilator-stimulated phosphoprotein (VASP), a protein implicated in the control of cytoskeletal dynamics and cell migration [40]. Ser239 is the major site of action PKG. Phosphorylation of VASP at Ser239 by PKG inhibits growth of vascular smooth muscle [41], in part, by capping actin filaments, resulting in filament retraction [42, 43]. The determination of P-VASP levels could be a novel indicator of both PKG-I activity and endothelium integrity under physiological and pathophysiological conditions in human tissue [11]. Our study suggested that VASP and P-VASP protein levels were depressed in IR model group, but elevated after being given SCU treatment, which was accompanied with the PKG-I fluctuation and the change of EtD of BA. Our result showed that the actions of PKG-I/VASP signaling may be a novel therapy target for IR injury of BA.

In summary, SCU produces a marked pharmacologic action and regulates cellular function through multiple pathways. Our present study firstly provides important evidence that SCU induced expression and activation of PKG-I and increased VASP expression in normal cultured and HR treated HBMECs. In CIR rats, SCU treatment decreased cerebral infarct size and augmented the endothelium-dependent relaxation in isolated BA against EtD caused by CIR, while the protective effects of SCU could be reversed by PKG inhibitor. These suggest that SCU protects endothelial cells against HR injury and improves endothelium-dependent relaxation which involves PKG/VASP signaling pathway. Our studies provide a new mechanism to explain cerebral protective effects of SCU.

## Figures and Tables

**Table 1:** Effect of SCU and PKG inhibitor administrations on ACh EC_50_ and *E*_max⁡_ in BA rings.

Group	Dosage (i.v.)	*n*	EC_50_ (*µ*M)	*E*_max⁡_ (% of U46619)
Sham	NS	12	0.39 ± 0.14	79.06 ± 6.57^*∗∗*^
Model	NS	11	1.11 ± 0.47	36.46 ± 4.90^##^
SCU	45 mg/kg	8	NA	22.47 ± 7.59^#^
SCU	90 mg/kg	11	0.30 ± 0.17	64.33 ± 4.40^*∗∗*^
PKG inhibitor	50 *μ*g/kg	8	NA	25.24 ± 7.19^#^
SCU + PKG inhibitor	90 mg/kg + 50 *μ*g/kg	15	NA	15.1 ± 4.90^*∗*#^

Data are means ± SEM; PKG inhibitor: Rp-8-Br-cGMPS; *E*_max⁡_ is the maximum vasodilative effect that ACh caused; EC_50_ is the ACh concentration that makes 50% of the maximum vasodilative effect. *n*: BA segments' number; in each group blood vessel rings were obtained from 3-4 rats with different treatments. Blood vessel rings in each group were pretreated by tail intravenous injection with SCU or NS; NA: not available as relaxative response is too weak to be tested; NS: normal saline; one-way ANOVA on Rank Kruskal-Wallis test, *E*_max⁡_ versus model, ^*∗*^ *P* < 0.05; ^*∗∗*^ *P* < 0.01, versus SCU (90 mg/kg), ^#^ *P* < 0.05, ^##^ *P* < 0.01.

## Abbreviations

SCU: Scutellarin
HR: Hypoxia reoxygenation
CIR: Cerebral ischemia reperfusion
IR: Ischemia reperfusion
PKG: cGMP-dependent protein kinase
EtD: Endothelial dysfunction
BA: Basilar artery
ACh: Acetylcholine
HBMECs: Human brain microvascular endothelial cells
p-VASP: Phosphorylated VASP
VASP: Vasodilator stimulated phosphoprotein.
